# Factors affecting quality of life in rectal cancer survivors who have undergone laparoscopic surgery: patient-reported outcomes over 10 years at a single institution

**DOI:** 10.1308/rcsann.2022.0156

**Published:** 2023-02-07

**Authors:** R Singh, D White, G Romano, E Osenda, S Allen, M Dunstan, R Elangovan, I Jourdan, T Rockall, A Scala

**Affiliations:** Royal Surrey NHS Foundation Trust, UK

**Keywords:** Rectal cancer, Laparoscopy, Total Mesorectal Excision (TME), PROMS, QOL

## Abstract

**Introduction:**

Colorectal cancer survivors have many problems affecting their quality of life (QOL). Traditional follow-up focuses on the detection of recurrence rather than QOL. Efforts are being made to assess patient-reported outcomes (PROMS) more formally. Such changes may enable patients to consider QOL factors when deciding on treatment.

**Methods:**

Patients who underwent laparoscopic surgery for rectal cancer between 2005 and 2015 at a single institution were identified and sent European Organisation for Research and Treatment of Cancer (EORTC) QLQ-C30 and QLQ-CR29 QOL questionnaires. QOL and the impact of radiotherapy, chemotherapy and formation of end colostomy were assessed.

**Results:**

Some 141 patients were identified: 12 died and 118 (83.7%) responded, of whom 101 completed the questionnaires and 17 declined to participate; 11 were lost to follow-up. Mean age was 67 years, median follow-up was 58 months. Median QOL score was 6 (maximum 7) and 4.5% of patients reported a poor QOL score (<4). Significant rectal/perianal pain, sexual dysfunction and urinary symptoms were reported in 3.6%, 10.9% and 2.7% of respondents, respectively. Significant differences between treatment groups were uncommon. All cohorts reported similar QOL, functional and symptom scores.

**Conclusions:**

These results compare favourably with the published data. Future studies may benefit from baseline assessment to better assess treatment impact, prescient in an increasingly elderly and comorbid population. This paper establishes that good PROMs are achievable with laparoscopic surgery for rectal cancer. It identifies limited differences in QOL between treatment modalities. Restoration of intestinal continuity and end colostomy result in similar QOL. This may address common concerns regarding stomata, sexual function and low anterior resection syndrome in this cohort.

## Introduction

Colorectal cancer survivors suffer many problems affecting quality of life (QOL), ranging from psychological distress to urological, bowel and sexual dysfunction.^1^ Follow-up programmes focus mainly on the detection of recurrence rather than QOL assessment.

There is a move to rebalance this to assess QOL robustly. The purpose is threefold: first, to provide patients with necessary support; second, to collate a body of evidence such that patients incorporate QOL in decisions regarding management strategies; and third, evidence is emerging that use of patient-reported outcome measures (PROMS) may improve survival in cancer patients.^2^ The National Institute of Health and Care Excellence now recommends PROMS be used as secondary endpoints in future clinical trials.^3^

There has been much work in identifying the best tools to assess QOL in colorectal cancer, much of it focusing on PROMS.^4^ There is, however, significant heterogeneity. Mcnair reported 53 different PROMs tools from 104 different papers.^5^ At present no consensus on a set of PROMs to assess QOL in survivors of colorectal cancer exists.

This paper reports PROMs from patients who have undergone laparoscopic surgery for rectal cancer at a single institution, utilising two validated instruments for QOL assessment. Both have been agreed on by a recent international collaborative aiming to incorporate PROMS in follow-up regimens.^4^

## Methods

A database of patients who have undergone surgical treatment for rectal cancer is kept at the Royal Surrey County Hospital. All patients treated from 2005 to 2015 were identified.

Information includes date of operation, operation type, complications, utilisation of chemotherapy or radiotherapy and date of last follow-up.

All patients were provided with a survey composed of the European Organisation for Research and Treatment of Cancer (EORTC) QLQ-CR29 and QLQ-C30 questionnaires. These PROMS tools were identified by the International Consortium for Health Outcomes Measurement’s Delphi exercise regarding patient-centred outcomes for colorectal cancer.^4^ This exercise was conducted over a period of 8 months culminating in February 2016.

The EORTC QLQ-C30 is a validated tool for measuring overall QOL in cancer patients. It is a combination of functional, symptom and global QOL scales. The QLQ-CR29 is a disease-specific module for colorectal cancer, created by the EORTC to supplement the QLQ-CR30. It comprises scales concerning bowel function, sexual function and gastrointestinal symptoms, and contains a stoma-specific component.

Comparisons were made between the following cohorts:

• above and below 36 and 60 months’ follow-up;
• the presence or not of a stoma;
• whether the patient had undergone radiotherapy;
• whether the patient had undergone chemotherapy;
• whether the patient had suffered a complication;
• above and below the median age;
• whether the patient had undergone anterior resection (AR) or abdominoperineal excision of the rectum (APR).

### Data analysis

Patients were initially contacted by post. Those who did not respond were then contacted by telephone and offered a repeat postal questionnaire or the opportunity to complete the survey by telephone. Results were tabulated electronically and data transformed as per the EORTC QLQ-C30 scoring manual. The same formulae were used and applied to QLQ-CR29, which has no formal scoring manual.

### Statistical analysis

For each variable, independent *t*-tests were performed to compare the means between groups; for example, APR vs AR. Levene’s test for homogeneity of variance was then performed. Statistical significance for both the homogeneity of variance and equality of means *t*-test was defined as *p*<0.05. For continuous variables such as age, the median value of the data set was taken as the cut-off point. All data were analysed using IBM SPSS version 23. For scale data, no composite values were used such as repeat values or means. If data were unavailable or missing they were excluded from the analysis.

## Results

Some 141 patients were identified: 12 patients died and 11 were lost to follow-up; 118 (83.7%) responded, of whom 17 refused to take part and 101 completed the questionnaires (71.6%). Sixty-nine respondents answered by post and 32 by telephone.

The mean age was 67 (47–88) years; the median follow-up 58 (21–162) months. There were 34 female and 67 male participants. Thirty-three patients had a tumour height below 5cm, 33 had a tumour height between 5 and 10cm, and 32 had a tumour height between 10 and 15cm. Tumour height was unknown for three participants.

Thirty patients underwent laparoscopic APR and 71 underwent a laparoscopic AR. Of the patients undergoing AR, 52 had a covering ileostomy formed, 50 of which were successfully reversed. One patient required formation of an end colostomy after reversal owing to an anastomotic leak. A second was given a colostomy 5 months postoperatively for rectal prolapse. No patients undergoing AR required end colostomy at their initial operation. Details regarding treatment modalities are shown in Table 1.

**Table 1:** Breakdown of patients by treatment modalities

Treatment Modality	*n*
Surgery alone	37
Preoperative chemotherapy	35
Preoperative radiotherapy	33
Postoperative chemotherapy	44
Preoperative chemotherapy and radiotherapy	31
Preoperative radiotherapy and postoperative chemotherapy	18

Complications occurred in 18 patients (18.8%). A list of complications is provided in Table 2.

**Table 2:** Complications

Complication	*n*
Anastomotic leak	8 (6 defunctioned, 2 managed conservatively)
Pulmonary embolus	2
High-output stoma	2
Bleed requiring transfusion	1
Perineal hernia	1
Rectovesical fistula	1
Cardiac arrest, return of spontaneous circulation	1
Adhesional small bowel obstruction	1
Anastomotic stricture	1

### EORTC QLQ-C30

All respondents answered all 30 questions. This questionnaire consists of five functional scales (physical, cognitive, role, emotional and social), three symptom scales (fatigue, pain, nausea and vomiting) and six individual items (dyspnoea, insomnia, loss of appetite, diarrhoea, constipation and financial difficulties), giving a total of 14 domains. Each of the scales comprises between two and five component questions. Functional scale scores are produced as a percentage, with a higher value denoting a better functional outcome. Symptom scales, again as a percentage, denote worse symptomatology. The symptom scales are reversed when calculating the global health score to allow for this difference, resulting in a higher score correlating with an improved global status or satisfaction.

There are two further questions assessing overall health and QOL, termed ‘Global’ questions. Detailed analysis of QOL score by treatment modality can be found in Appendix 1 (available online) but are summarised below. For other factors such as age, time from follow-up and complications, detailed QOL analysis is shown in Appendix 2 (available online).

#### Overall health

Question 29 asks patients to rate their overall quality of health and question 30 their overall QOL. Patients respond on a scale of 1–7, with 1 pertaining to worse health and 7 to perfect health. The modal score for both questions was 6. Some 48% of responders answered question 29 with a score of 6 or 7 and 61% of patients answered question 30 with a score of 6 or 7.

#### AR vs APR

APR patients reported better scores for diarrhoeal symptoms, this being the only statistically significant difference. Patients undergoing APR reported better functional and symptom scores in nearly all categories, fairing slightly worse in terms of loss of appetite and pain. They also reported better overall health and QOL scores. The results did not reach statistical significance.

#### Chemotherapy

Patients who had undergone chemotherapy reported better global (overall health and overall QOL) scores, reaching statistical significance (*p* < 0.05). They also tended to report better functional scores in each of the five domains. Chemotherapy patients reported worse symptom-related scores, except for nausea, vomiting and constipation, and reported more problems with financial difficulties. These results were not statistically significant.

#### Radiotherapy

Patients who had undergone radiotherapy reported better scores in terms of physical function, role function and cognitive function. Worse scores were reported for emotional and social function. Radiotherapy patients reported worse scores for most symptom areas, except for nausea and vomiting, dyspnoea and constipation. Global scores were higher in the radiotherapy group. No results in this comparison achieved statistical significance.

#### Stomata

Thirty-seven patients had a stoma at the time of data collection; 64 did not. There were no statistically significant differences found between patients with or without stomata. Excluding fatigue and pain, stoma patients reported better scores across all categories, and reported a positive overall QOL. Some 5% of patients graded QOL below the middle value of 4, and 65% assigned QOL a score of 6 or 7.

#### Length of follow-up

At the time of data collection, 87 patients were more than 36 months from their operation and 14 patients were less than 36 months from their operation. No statistically significant differences were seen between both groups. There was a trend towards improved QOL scores in patients who were less than 36 months from their operation. This cohort almost uniformly reported better functional, symptom and global QOL scores. The exception was for shortness of breath.

Sixty-three patients were more than 60 months from their operation and 38 patients less than 60 months from their operation. No results reached statistical significance when comparing both groups. Patients more than 60 months from their operation reported better functional scores, except for emotional function. Symptom scores were split almost equally between the groups. Global scores tended to be higher in patients who were less than 60 months from their operation.

#### Other factors

Patients who suffered complications did not routinely report worse functional, symptom or global scores. When compared with patients who did not suffer a complication, there were no statistically significant results.

In terms of age, patients above and below the mean age answered similarly. The older group did, however, report more positive scores that were statistically significant for physical, emotional and cognitive function.

### EORTC QLQ-CR29

The EORTC QLQ-CR29 is a disease-specific module designed to be used in conjunction with EORTC QLQ-CR30. It contains 29 questions. There are 4 functional and 18 symptom scales. Four scales comprise two questions, the remainder comprise one question.

Of the 101 returned questionnaires, 25 patients did not populate the survey completely. Of these, the majority did not answer one or both questions regarding sexual function. Given these symptom scales involved a maximum of two data points, accounting for them in data analysis would result in significant potential for bias. These 25 patients were therefore been excluded from the analysis.

#### AR vs APR

Fifty-four patients underwent AR and 22 patients APR. APR patients reported higher scores for sexual interest. AR patients reported higher scores for sexual difficulties. Both results were statistically significant. There were no other statistically significant results, and there was an even spread of reported outcomes between groups.

#### Chemotherapy

Forty-four patients underwent chemotherapy and 32 patients did not. Chemotherapy patients reported worse scores for dysuria, problems with taste and sore skin, all results being statistically significant. There were no other statistically significant results. Chemotherapy patients reported worse scores in 13 of 21 domains.

#### Radiotherapy

Twenty-eight patients underwent radiotherapy and 48 did not. Radiotherapy patients reported worse scores for faecal incontinence: this was statistically significant. No other results were statistically significant. Patients who had undergone radiotherapy reported worse scores in 16 of 21 domains.

#### Stomata

Twenty-nine patients had a stoma at the time of data collection, whereas 47 did not. Patients with a stoma reported higher scores for sexual interest. This was the only statistically significant result. Patients without a stoma reported worse scores in 12 of 20 domains. The question regarding stoma difficulties was excluded from this analysis because it was not comparable.

#### Length of follow-up

At the time of data collection, 66 patients were more than 36 months from their operation, whereas 10 patients were less than 36 months from their operation. Patients who were less than 36 months from their operation reported greater scores for anxiety and worries about weight. They also reported higher scores for sexual interest. These were all statistically significant results.

Forty-five patients were more than 60 months from their operation, whereas 31 patients were less than 60 months from their operation. There were three statistically significant differences. Patients less than 60 months from their operation reported more worries about weight, and patients who were more than 60 months from their operation reported worse symptoms with regards to bloating and abdominal pain.

Although not reaching statistical significance, patients more than 36 months from their operation reported worse scores in 14 of 21 domains. Patients more than 60 months from their operation reported worse scores in 13 of 21 domains.

#### Other factors

Fifteen patients suffered complications, whereas 61 did not. Patients with complications reported significantly worse scores for hair loss and mouth dryness. No other results were significant. Patients above and below the mean age reported similar scores.

## Discussion

This study has assessed PROMs for QOL following surgery for rectal cancer in a large cohort. The study population is heterogenous, including participants with permanent and temporary stomata, those who have had restoration of intestinal continuity, and those who have undergone chemotherapy and radiotherapy. Patients have been compared who have undergone different treatment modalities and vary widely in terms of age and stage of recovery. PROMs have been assessed using validated measurements for their condition.

The questionnaires cover 59 symptom- and function-related areas. Considering this large number and broad range of questions, statistically significant differences between groups are few. They are summarised in Table 3. The relatively low number of statistically significant differences between comparative groups is in line with several published papers on the subject.

**Table 3:** Statistically significant results by questionnaire and comparator group

EORTC QLQ-C30
APR patients reported better scores for diarrhoeal symptoms
Chemotherapy patients reported better overall health and overall quality-of-life scores
Patients above the mean age reported more positive scores for physical, emotional and cognitive function
EORTC QLQ-CR29
APR patients reported higher scores for sexual interest
AR patients reported higher scores for sexual difficulties
Chemotherapy patients reported worse scores for dysuria, problems with taste and sore skin
Radiotherapy patients reported worse scores for faecal incontinence
Patients with a stoma reported higher scores for sexual interest
Patients more than 36 months from their operation reported greater scores for worries about weight and health
Patients more than 36 months from their operation reported higher scores for sexual interest
Patients less than 60 months from their operation reported more worries about weight
Patients more than 60 months from their operation reported worse symptoms with regards to bloating and abdominal pain
Patients who suffered complications reported worse scores for hair loss and mouth dryness

APR = abdominoperineal excision of the rectum; AR = anterior resection

Several areas showed a trend towards significance, which may have been more marked given a larger sample size. A baseline questionnaire would have been beneficial in this instance, because many symptoms may have predated surgery in this elderly cohort. This should be a necessary line of enquiry for future prospective studies.

One striking finding is that patients with a permanent stoma report similar QOL to those who have had intestinal continuity restored. Questions regarding sexual functioning were better in permanent stoma patients in particular, in contrast to several other papers.^6-8^ Although they reported different sets of problems **–** for example, AR patients reported worse symptoms regarding diarrhoea, and APR patients regarding embarrassment – overall QOL scores were similar.

Although data exist to suggest that a stoma has an appreciably negative impact on quality of health,^7,9,10^ there is contrasting evidence consistent with our cohort’s results. Camilleri-Brennan *et al* arrived at similar conclusions in their prospective, case-matched comparison of APR and AR patients,^6^ as did Gosselink.^9^ A Cochrane review published in 2012 also concluded that QOL in both groups of patients is similar.^11^ This is perhaps a reflection of the quality of support for stoma patients, and may also be an example of ‘reframing’, where acceptance of certain consequences of treatment leads to an improved perception of QOL.^12,13^ Stoma patients have consistently reported better scores for symptoms relating to diarrhoea and gastrointestinal function in a number of studies.^8,14,15^

Higher overall scores for older patients in physical, emotional and cognitive function may also be an example of reframing. This matches the findings of Arndt *et al*, who reasoned they may accept worse function as part of increasing age, both in the context of having undergone multimodal cancer therapy and in comparing overall health with peers. Furthermore, younger patients may not have access to the same resources that can help as coping strategies.^16^

The effect of adjuvant therapy was not as marked as expected, although patients who had undergone radiotherapy in particular tended to report worse scores, reaching statistical significance for diarrhoea. Chemotherapy patients reported better overall QOL, but they reported worse symptom-related scores. Although not statistically significant in the majority of cases, one can infer from the trends that adjuvant therapy negatively impacts patients far more than the modality of surgery. Much other data support this.^8,17-19^

In terms of overall quality of health and life, our cohort compare well with other rectal cancer patients. This can, however, be difficult to compare directly because few papers use the same combination of questionnaires. Some 48% and 61% of responders in our study replied with a score of 6 or 7 to questions 29 and 30 in the EORTC QLQ-C30 questionnaire, respectively; corresponding to good or perfect health and QOL. A UK Department of Health report in 2015 that included over 6,000 rectal cancer patients described that 29% reported perfect health, compared with 40% of the general population.^1^

This paper is subject to various limitations. The data were limited to patients who chose to respond. This may mean those with potentially significantly different experiences were not assessed. Patients answered either by post or by telephone; it is possible that two different methods of populating the questionnaire influenced the responses given. Patients who had filled out EORTC QLQ-CR29 incompletely were excluded from the analysis, which may also have exerted bias. The study was not designed or powered to assess for one clinical or statistical endpoint. The paper did not consider concurrent medical illness, or level of anastomosis, both which have shown to adversely affect QOL.^20-23^

The study does not contain a comparator for pretreatment QOL. Although we have shown that overall QOL is good post treatment, we have not illustrated how it has changed as a result of the various therapies employed. Similarly, we have assessed QOL at one time point postoperatively, with all participants responding at various lengths of time post treatment. A series of assessments of QOL would have been beneficial in this respect with questionnaires at predefined intervals: for example, at baseline, 3, 6 and 12 months postoperatively, and yearly thereafter. Patients with early tumours managed with endoluminal approaches have also not been assessed because the primary focus of the paper was to assess the impact of resectional surgery. This is an area of increasing importance and such approaches may have a significantly lower impact on QOL. These limitations are being addressed by a prospective study (the CiTRus trial) currently underway at the Royal Surrey County and Clatterbridge hospitals (ClinicalTrials.gov Identifier: NCT04697394).

Although these limitations should qualify interpretation of the data, the authors feel, after assessment of other studies, that the sample size and methodology mean that important conclusions can be drawn.

QOL and PROMs are increasingly being incorporated in colorectal cancer management and follow-up programmes. Work is currently underway to assess whether patient- rather than clinician-led follow-up may have an impact on clinical outcomes.^24^

## Conclusions

Our paper has shown that overall QOL is high after surgical treatment for rectal cancer, but specific factors may influence different symptoms. More work that uses standardised PROM and QOL assessment measures is required, such that patients can be better informed regarding functional and symptomatic issues that may occur following treatment. This is particularly important in early-stage disease, where a variety of organ-preserving strategies are now available. Ultimately, this will guide better patient-centred decision-making in rectal cancer and assist in identifying and delivering tailored solutions to QOL issues in the postoperative setting.

## References

[1] UK Department of Health. Quality of life of colorectal cancer survivors in England. 179. https://www.england.nhs.uk/wp-content/uploads/2015/03/colorectal-cancer-proms-report-140314.pdf (cited December 2023).

[2] Basch E, Deal AM, Dueck AC *et al.* Overall survival results of a trial assessing patient-reported outcomes for symptom monitoring during routine cancer treatment. *JAMA* 2017; **318**: 197. 28586821 10.1001/jama.2017.7156 PMC5817466

[3] National Institute for Health Care and Excellence (NICE). *Colorectal cancer: diagnosis and management* Guidance and guidelines NG151. https://www.nice.org.uk/guidance/ng151. (cited December 2023).

[4] Zerillo JA, Schouwenburg MG, van Bommel ACM *et al.* An international collaborative standardizing a comprehensive patient-centered outcomes measurement set for colorectal cancer. *JAMA Oncol* 2017; **3**: 686. 28384684 10.1001/jamaoncol.2017.0417

[5] Mcnair AGK, Whistance RN, Forsythe RO *et al.* Synthesis and summary of patient-reported outcome measures to inform the development of a core outcome set in colorectal cancer surgery. *Color Dis* 2015; **17**: O217–O229. 10.1111/codi.13021 PMC4744711 26058878

[6] Camilleri-Brennan J, Steele RJC. Objective assessment of morbidity and quality of life after surgery for low rectal cancer. *Color Dis* 2002; **4**: 61–66. 10.1046/j.1463-1318.2002.00300.x 12780658

[7] Engel J, Kerr J, Schlesinger-Raab A *et al.* Quality of life in rectal cancer patients. *Ann Surg* 2003; **238**: 203–213. 12894013 10.1097/01.sla.0000080823.38569.b0 PMC1422675

[8] Guren MG, Eriksen MT, Wiig JN *et al.* Quality of life and functional outcome following anterior or abdominoperineal resection for rectal cancer. *Eur J Surg Oncol* 2005; **31**: 735–742. 16180267 10.1016/j.ejso.2005.05.004

[9] Gosselink MP, Busschbach JJ, Dijkhuis CM *et al.* Quality of life after total mesorectal excision for rectal cancer. *Color Dis* 2006; **8**: 15–22. 10.1111/j.1463-1318.2005.00836.x 16519633

[10] Hoerske C, Weber K, Goehl J *et al.* Long-term outcomes and quality of life after rectal carcinoma surgery. *Br J Surg* 2010; **97**: 1295–1303. 20602501 10.1002/bjs.7105

[11] Pachler J, Wille-Jørgensen P. Quality of life after rectal resection for cancer, with or without permanent colostomy. *Cochrane Database Syst Rev* 2012. 10.1002/14651858.CD004323.pub4 PMC7197443 23235607

[12] Anthony T, Jones C, Antoine J *et al.* The effect of treatment for colorectal cancer on long-term health-related quality of life. *Ann Surg Oncol* 2001; **8**: 44–49. 11206224 10.1007/s10434-001-0044-2

[13] Calandra DM, Di Mauro D, Cutugno F, Di Martino S. Navigating wall-sized displays with the gaze: A proposal for cultural heritage. *CEUR Workshop Proc* 2016; **1621**: 36–43.

[14] Bloemen JG, Visschers RGJ, Truin W *et al.* Long-term quality of life in patients with rectal cancer: association with severe postoperative complications and presence of a stoma. *Dis Colon Rectum* 2009; **52**: 1251–1258. 19571701 10.1007/DCR.0b013e3181a74322

[15] Fischer A, Tarantino I, Warschkow R *et al.* Is sphincter preservation reasonable in all patients with rectal cancer? *Int J Colorectal Dis* 2010; **25**: 425–432. 20127342 10.1007/s00384-010-0876-y

[16] Arndt V, Merx H, Stegmaier C *et al.* Restrictions in quality of life in colorectal cancer patients over three years after diagnosis: a population based study. *Eur J Cancer* 2006; **42**: 1848–1857. 16829069 10.1016/j.ejca.2006.01.059

[17] Varpe P, Huhtinen H, Rantala A *et al.* Quality of life after surgery for rectal cancer with special reference to pelvic floor dysfunction. *Color Dis* 2011; **13**: 399–405. 10.1111/j.1463-1318.2009.02165.x 20041930

[18] Smith-Gagen J, Cress RD, Dramke CM. Quality-of-life and surgical treatments for rectal cancer — a longitudinal analysis using the California Cancer Registry. *Psychooncology* 2010; **19**: 870–878. 10.1002/pon.1643 PMC2911491 19862692

[19] Thong MSY, Mols F, Lemmens VEPP *et al.* Impact of preoperative radiotherapy on general and disease-specific health status of rectal cancer survivors: a population-based study. *Int J Radiat Oncol Biol Phys* 2011; **81**: 49–58. 10.1016/j.ijrobp.2010.12.030 21362582

[20] Allal AS, Gervaz P, Gertsch P *et al.* Assessment of quality of life in patients with rectal cancer treated by preoperative radiotherapy: a longitudinal prospective study. *Int J Radiat Oncol Biol Phys* 2005; **61**: 1129–1135. 15752893 10.1016/j.ijrobp.2004.07.726

[21] Kuzu MA, Topçu Ö, Uçar K *et al.* Effect of sphincter-sacrificing surgery for rectal carcinoma on quality of life in Muslim patients. *Dis Colon Rectum* 2002; **45**: 1359–1366. 12394435 10.1007/s10350-004-6425-4

[22] Grumann MM, Noack EM, Hoffmann IA, Schlag PM. Comparison of quality of life in patients undergoing abdominoperineal extirpation or anterior resection for rectal cancer. *Ann Surg* 2001; **233**: 149–156. 11176118 10.1097/00000658-200102000-00001 PMC1421194

[23] Kriegsman DMW, Deeg DJH, Stalman WAB. Comorbidity of somatic chronic diseases and decline in physical functioning: the longitudinal aging study Amsterdam. *J Clin Epidemiol* 2004; **57**: 55–65. 15019011 10.1016/S0895-4356(03)00258-0

[24] Hovdenak Jakobsen I, Juul T, Bernstein I *et al.* Follow-up after rectal cancer: developing and testing a novel patient-led follow-up program. Study protocol. *Acta Oncol (Madr)* 2017; **56**: 307–313. 10.1080/0284186X.2016.1267400 28068158

